# Netflow Python library – A free software tool for the generation and analysis of pore or flow networks

**DOI:** 10.1016/j.mex.2021.101592

**Published:** 2021-11-23

**Authors:** Daniel W. Meyer

**Affiliations:** Institute of Fluid Dynamics, ETH Zürich, Zürich, Switzerland

**Keywords:** Dendrogram, Cluster, Heterogeneity, Connectivity, Porous media, Pathway, Periodic, Unbounded, Digital rock analysis

## Abstract

State-of-the-art tomographic scanning techniques provide detailed pore-space geometries of natural porous media, which are central for the study of subsurface flow and transport. Due to experimental and computational limitations, the extraction of high-resolution images is limited to relatively small sample volumes. To reduce the amount of data and the physical complexity, pore-space geometries are routinely translated into pore network models. Subsequently, such networks are expanded in space with suitable computational methods to determine effective medium parameters at larger scales relevant in engineering applications. While existing methods can provide networks with effective flow parameters being consistent with experimental data for comparably homogeneous media such as bead packs and sandstones, these methods are inadequate for more complex heterogeneous rocks such as carbonates or become too expensive for large networks. The netflow Python library accompanying this paper extends existing methods by preserving pore clusters that are a key characteristic of heterogeneous rocks. To this end dendrograms are extracted from experimental data and perturbed when generating larger networks. Moreover, the methods included in the netflow library are implemented in computationally efficient ways and enable the generation of large periodic networks that virtually eliminate boundary effects, which interfere in existing methods.

• The netflow Python library enables the generation of large irregular networks, as it preserves pore or node clusters which are present in certain natural rock types.

• The netflow Python library allows for the generation and flow analysis of boundary-free periodic networks. It further includes methods to convert periodic networks into conventional cubical ones.

## Graphical abstract

**Figure:**
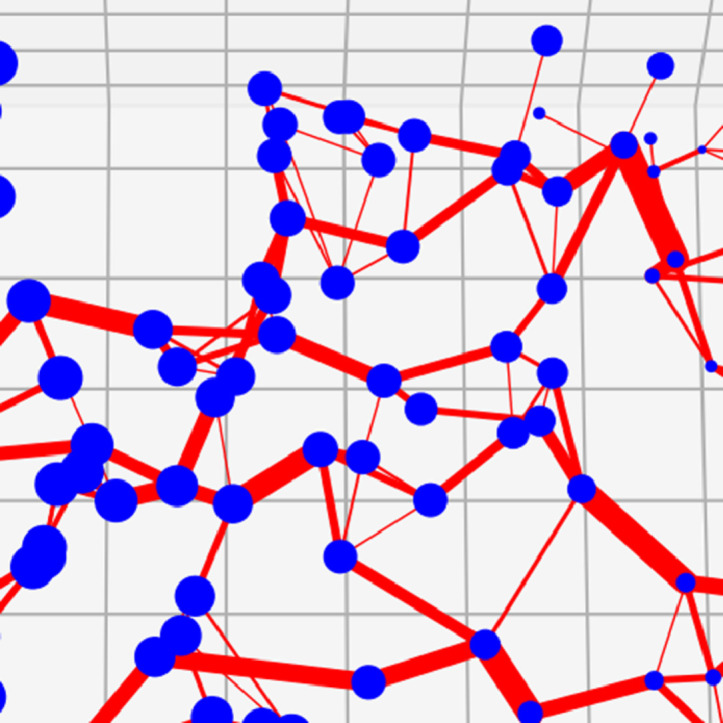

Specifications table

**Table:** 

Subject Area	Earth and Planetary Sciences
More specific subject area	Geology, Subsurface Hydrology
Method name	Generation of large-scale pore or flow networks
Name and reference of original method	Idowu, N.A., Blunt, M.J. Pore-Scale Modelling of Rate Effects in Waterflooding.Transp Porous Med 83, 151–169 (2010). https://doi.org/10.1007/s11242-009-9468-0
Resource availability	Method is contained in the supplemental material to this paper.

*Method details

## Library overview and structure

In the netflow library, an object-oriented programming approach has been followed that enables compact implementations and intuitive use. The library relies besides a few standard Python components on the h5py package (hdf5 file support) and the PyAMG library (algebraic multi-grid solver for flow solutions) and is composed of the following two modules:

*netgen.py* contains functionality to generate and manipulate pore or flow networks from an existing (typically smaller) base network. The generated networks are periodic in space and as such do not suffer from boundary effects in comparison to bounded networks produced by existing methods [e.g., 1]. This is because pores or network nodes are subject to the same conditions irrespective of whether they are located inside the network domain or close to domain boundaries. With periodic networks, the only limitation in comparison to infinite networks is the fact that the largest scale of representation is limited by the domain size aka the period. In case pore or node clusters are present in the base network, a dendrogram-based generator is available that preserves clustering. For the connection of pores with throats, the pore neighborhood needs to be explored in a computationally efficient way to avoid size-limitations of existing methods [e.g., 1]. To this end, the netflow implementations rely on an optimized Delaunay triangulation being part of the SciPy library. Algorithmic details about the construction of periodic throat connections are either available in the code or are provided in one of our earlier contributions (section 2.1 in [2]) or (steps 5 and 6 on page 3 of [3]).

The second module *netflow.py* includes functions to determine flow and pressure distributions in bounded and periodic networks. Here, we make use of the algebraic multi-grid solver provided through the PyAMG library. The netflow library has been applied to compile the results contained in [2] and it has been compared against method [1] in our companion paper [3], where its computational efficiency has been documented as well. The following table contains most of the functions and objects thatcomprise the netflow library.

**Table:** 

Functions for …	
network generation	generate_imperial, generate_dendrogram, generate_simple_net
network manipulation	cut_network, erase_network, open_periodic_network
flow analysis	solve_flow_inout, solve_flow_periodic, flux_balance, flux_plane
in-/output and visualization	plot_network, imperial_read, load_network_from, load_flow_from, save_network_to, save_flow_to
Objects	with properties
Network Pore Throat	label, pores, throats, lb, ub label, throats, pos, r label, pore1, pore2, r

The most important methods are illustrated in the following examples. To reproduce the examples below and for a more complete documentation of the netflow functions and objects, the reader is referred to the jupyter notebook, which is included in the supplemental material together with the library source code and network-data.

## Basics

### Load and inspect a demo network

Pore networks can be imported through the function *imperial_read* from text files available in the Imperial College network format defined in [4]. Alternatively, a network that was previously stored to a hdf5 file with *save_network_to* can be imported with *load_network_from*. Below, the demo network contained in the supplemental material is imported from an hdf5 file and subsequently plotted:

In:

import matplotlib.pyplot as plt

from netflow import *

# load demo network

demonet = netflow.load_network_from('network.h5′)

_ = netflow.plot_network(demonet)

Out:

**Figure:**
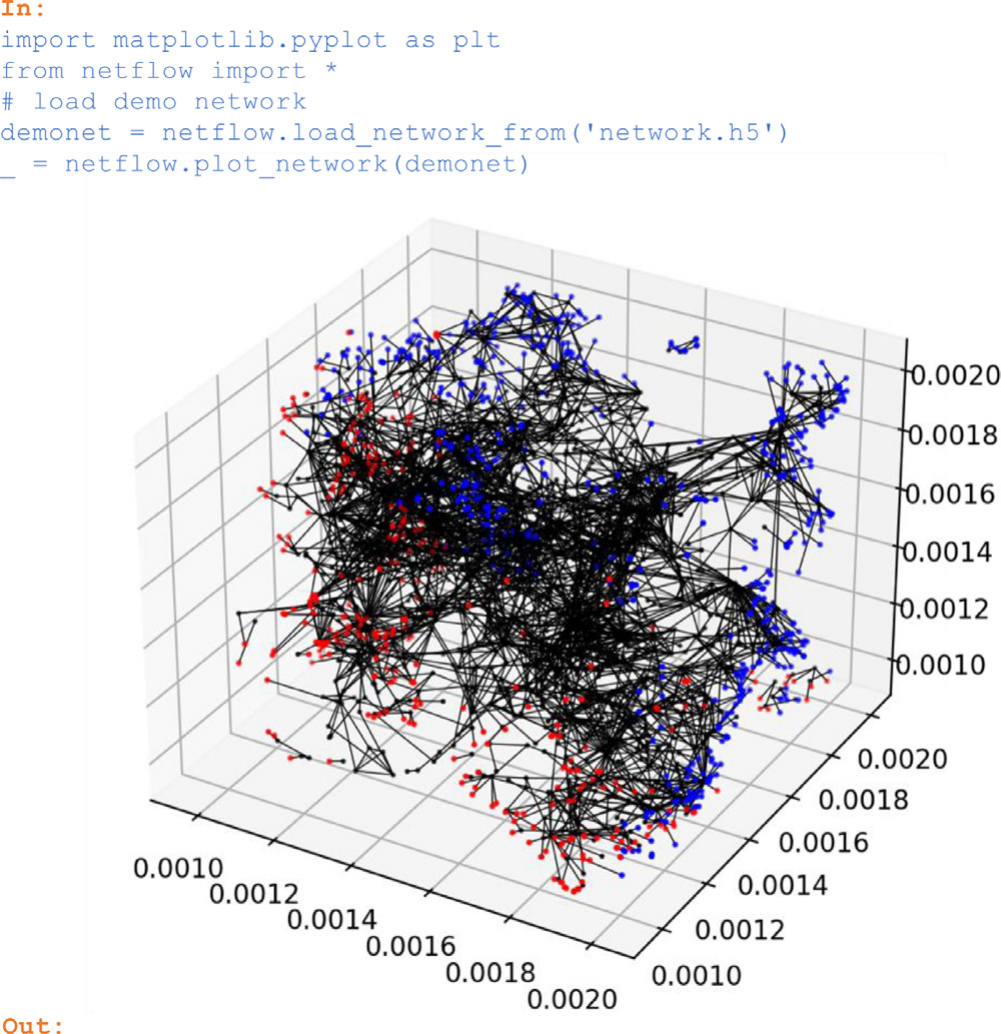

### Network data structure and objects

The network data is structured by means of a network object that contains sets with pore and throat objects. Moreover, a network object can be tagged with the string *label* and the spatial extension of the cubical network domain is set by the properties *lb* and *ub*. These two variables store lists with the coordinates of the lower resp. upper bounds. Except for the function *plot_network*, which is limited to 3d networks, all implementations can accommodate 3d as well as 2d networks. A network object has methods *add_pore*/*remove_pore* and *connect_pores*. Besides adding/removing pores resp. connecting them with throats, these methods update the network's pore and throat lists.

In the case of a pore, the *label* string can be used to identify pore groups serving, e.g., as in-/outlets or having resulted from a network cutting operation as shown later in section 4.2. Labels of throats on the other hand are used to describe periodic throats that connect pores at the opposite network domain faces. Documentation strings are available for most library elements. For example, labels of periodic throats are documented in the throat class:

In:

print(netflow.Throat.__doc__)

Out:

Class of a throat connecting two pores.

Periodic throats have a label 'X1 X2 × 3′ with Xc being element of {-1,0,1}.

For Xc = 1, pore1 is at the right or upper domain bound in the c-direction

and pore2 is at the left or lower bound. Vice versa for Xc = -1. With

Xc = 0, the throat does not cross the bound periodically in c-direction.

## Generation of periodic cubical networks

Different random network generation algorithms are implemented that produce cubical networks with periodic domain boundaries.

### Networks with uniformly-distributed pores

The method *generate_simple_net* produces networks with *n_pores* uniformly-distributed pores with radius *r_pore* that are connected to its closest neighbors through *coordinatnumb* throats of radius *r_throat*. To identify neighboring pores, the computationally efficient implementation of the Delaunay triangulation, which is part of the SciPy library, is used in *generate_simple_net* and in the other generators contained in the netflow library. In *generate_simple_net*, the argument *sd* is the seed of the random number generator that can be left random (set to *None*) or that can be prescribed to generate networks under reproducible conditions.

In:

simplenet = netgen.generate_simple_net(n_pores=1e2, targetsize=[2,1.5,0.5], r_pore=0.01,

r_throat=0.001, coordinatnumb=4, Lmax=0.45, sd=1)

_ = netgen.plot_network(simplenet)

Out:

**Figure:**
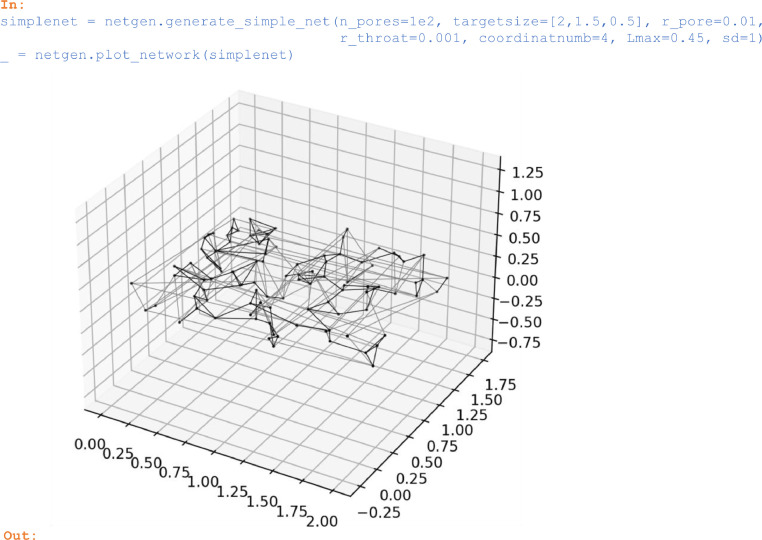

Periodic network throats that connect pores located at the opposite faces of the network domain were plotted with light gray lines in the above figure. While in the plot these connections traverse the domain, in fact they cross domain faces and connect to pores located in neighboring periodic copies of the network.

The network generation algorithm outlined by Idowu and Blunt [1] is implemented in the function *generate_imperial*. Their method is based on a uniform pore distribution and accounts for the throat statistics of an existing base network. Compared to the original implementation in [1], which leads to impractically high generation times for increasing pore counts, the present Python library applies a computationally efficient triangulation-based pore-neighbor search and generates periodic networks. While method [1] works well for bead packs and sandstones, it is not suited for irregular pore networks encountered for example in carbonate rocks [3].

### Networks with pore clusters

The dendrogram-based network generation algorithm introduced in our companion paper [3] accounts for throat statistics of a base network in a similar way as [1] and additionally preserves pore clusters. The latter are characterized by means of a dendrogram [5] and our according generator is implemented in *generate_dendrogram*. For illustration, a new network is generated from the demo network that was imported in section 2.1:

In:

dendronet = netgen.generate_dendrogram(basenet=demonet, targetsize=[1,1,1],

cutoff=0.5*(demonet.ub[0]-demonet.lb[0]),

sd=1, mute=True)

_ = netgen.plot_network(dendronet)

Out:

**Figure:**
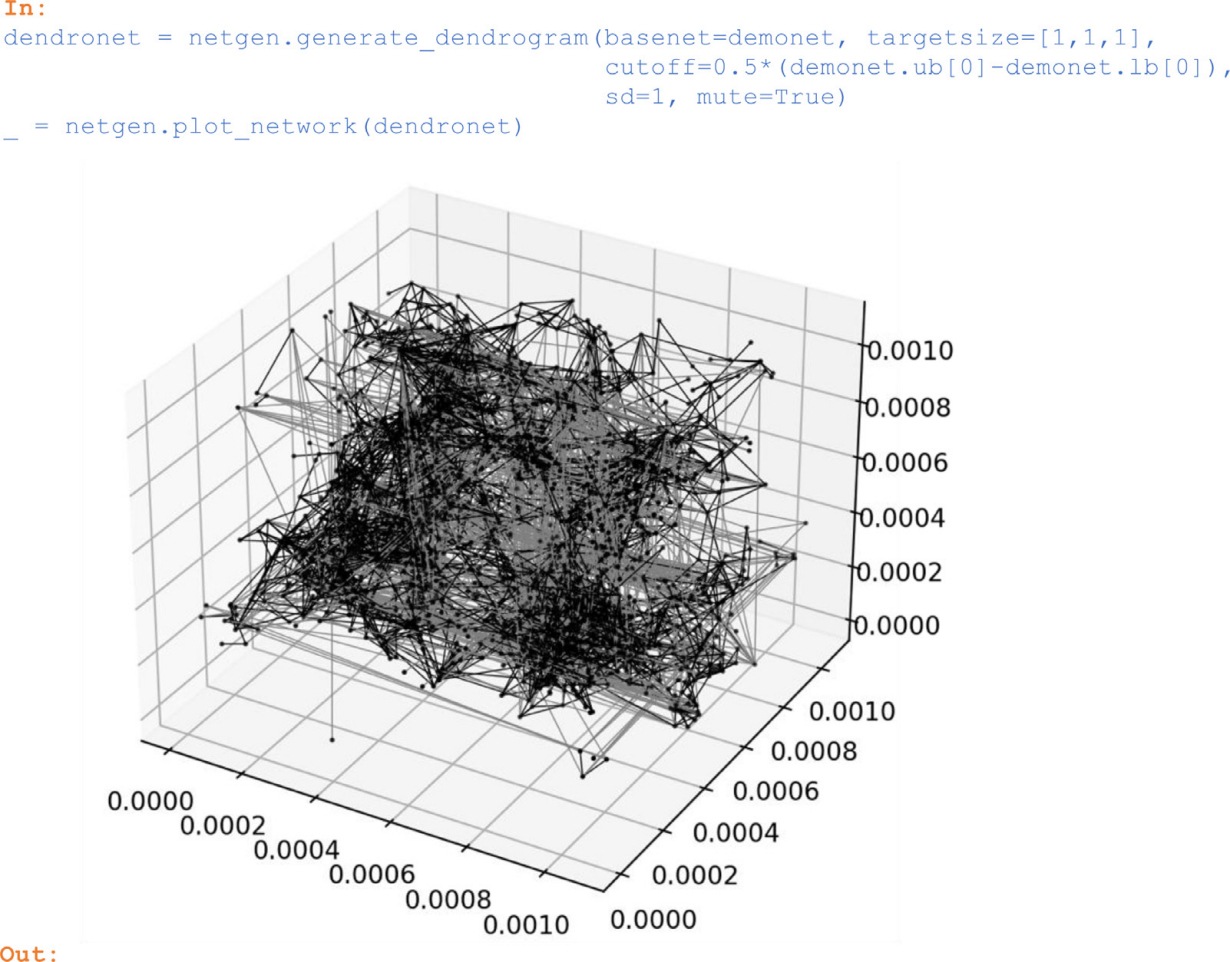

Unlike in *generate_simple_net*, where the *targetsize* argument specifies the physical extension of the generated network, in *generate_dendrogram* the physical extension results from multiplying the *targetsize* with the extension of the base network. When generating large networks within the Python interpreter, *generate_dendrogram* with *mute=False* will provide progress information. With the *cutoff* argument, the size of the randomly perturbed clusters can be limited. In the example above, clusters smaller than 50% of the base network size were perturbed. Setting *cutoff=0* will not perturb any of the clusters and retain the pore arrangement from the base network.

## Manipulate networks

Periodic networks have the advantage that they do not suffer from boundary effects. Nevertheless, in many applications bounded networks are eventually needed. Through the following operations, unbounded periodic networks can be transformed into conventional bounded ones.

### Opening periodic throat connections

Periodic throat connections crossing network domain faces that are normal to the spatial direction *c* (= 1, 2, or 3) can be opened by *open_periodic_network*. In the opening process of periodic throat connections, pores that originally reside inside the network domain are copied outside of the domain. These copies are referred to as in/out pores (copies beyond left resp. right domain boundary).

In:

inouts = {} # pore dict to avoid duplicate pore copies when opening periodic throats

netflow.open_periodic_network(simplenet, c=1, inouts=inouts)

netflow.open_periodic_network(simplenet, c=2, inouts=inouts)

_ = netgen.plot_network(simplenet)

Out:

**Figure:**
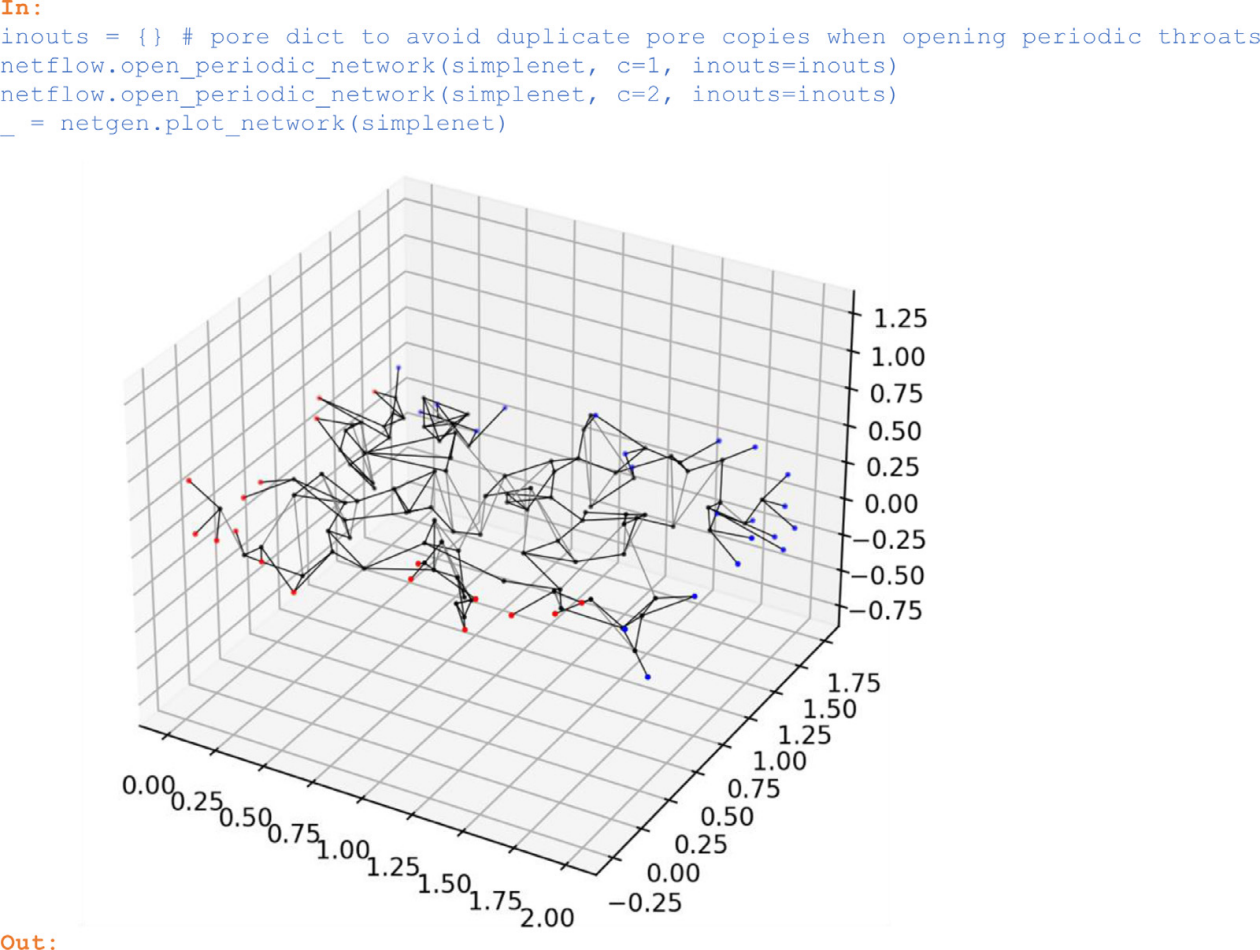

While opening periodic throat connections in the 1 and 2-directions, *open_periodic_network* generated 40 in/out pores as seen in the above plot (red resp. blue pores).

### Cutting networks

Planar network faces can be generated by applying a cutting plane to a network and by erasing the network section on one side of the cutting plane. When cutting a network with *cut_network*, pores with radii = 0 are introduced at the intersection points of throats and the cutting plane. The cutting plane is defined as being normal to the *c*-axis (with *c* = 1, 2, or 3) and located at position *x* along the *c*-axis. Before cutting, periodic throat connections in *c*-direction are opened by invoking *open_periodic_network*.

In:

netflow.cut_network(simplenet, x=0.5, c=1)

_ = netgen.plot_network(simplenet)

Out:

**Figure:**
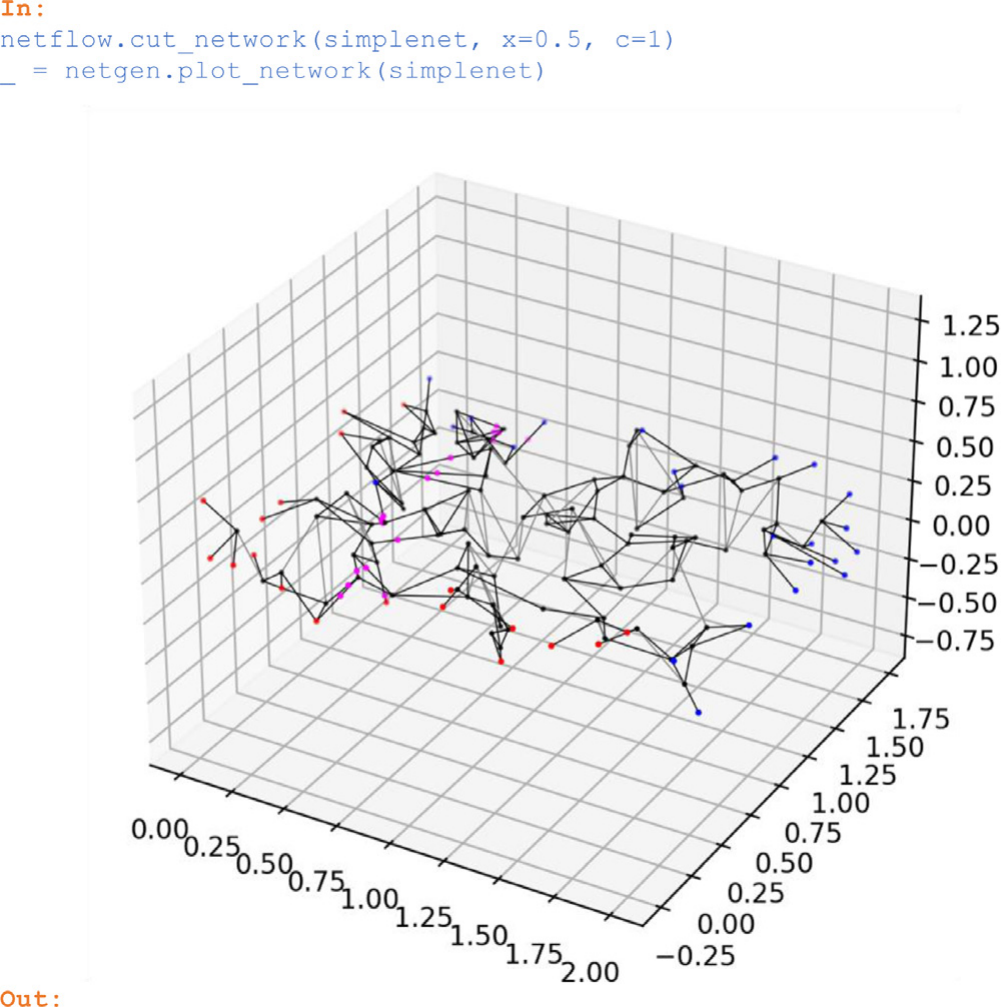

*plot_network* has rendered pores that resulted from *cut_network(simplenet, x=0.5, c=1, ...)* with magenta points.

### Erasing parts of a network

The method *erase_network* can be applied to remove pores that are located to the left or to the right of a point *x* on the *c*-axis. Throats that connect to these pores will be removed as well:

In:

netflow.erase_network(simplenet, x=0.5, c=1, direct=True, label='inlet')

_ = netgen.plot_network(simplenet)

Out:

**Figure:**
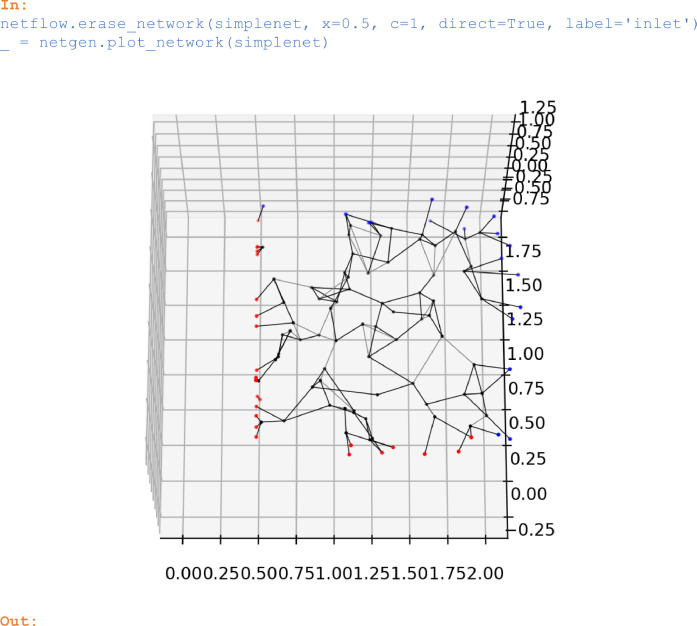

In connection with *cut_network(simplenet, x=0.5, c=1, ..., label='inlet')* from above, a network face at x_1_ = 0.5 parallel to the x_2_-x_3_ plane resulted. The pores on that face were labelled with *'inlet'*.

### Adding pores/throats

To connect for example the previously constructed pores with labels *'inlet'* to one single inlet pore, the following operations are needed:

In:

source = netflow.Pore(pos=[0,0.75,0.25], r=0, label='in1′) # single inlet pore

simplenet.add_pore(source)

# connect new source pore to pores labelled with 'inlet'

for pore in simplenet.pores:

if (pore.label == 'inlet'): # loop over inlet pores

pore.label = ''

simplenet.connect_pores(pore1=source, pore2=pore, r=0.05) # connect to source

# plotting

_ = netgen.plot_network(simplenet)

Out:

**Figure:**
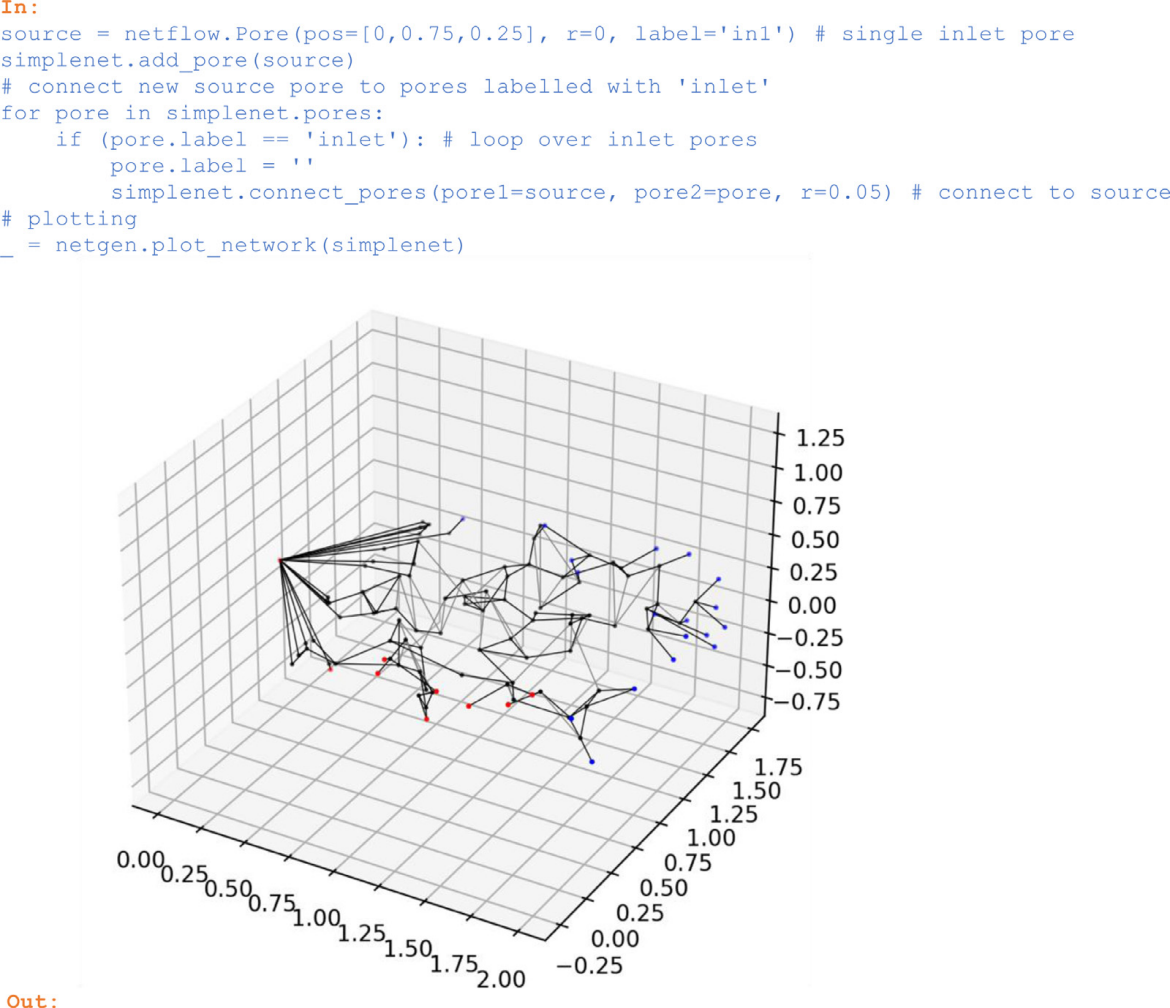

## Flow simulation

In the case of a non-periodic network with distinct sets of in- and outflow pores, the function *solve_flow_inout* can be applied where pressures p_in_ resp. p_out_ are prescribed and pore pressures and throat fluxes are returned.

In:

# solve flow

inpores = {source}

outpores = {pore for pore in simplenet.pores if pore.label == 'out1′}

(press,flux) = netflow.solve_flow_inout(simplenet, pin=1e4, pout=0, inpores=inpores,

outpores=outpores, mu=1e-3)

# plotting

ax = plt.figure().add_subplot(111, projection='3d')

# plot pressure bubbles (blue)

pnts = [pore.pos for pore in press]; pnts = list(map(list, zip(*pnts))) # transpose

size = [p/1e4 * 400 for pore, p in press.items()]

ax.scatter(*pnts, s=size, marker='.', color='b', depthshade=False)

# plot outlet pores (green)

pnts = [pore.pos for pore in outpores]; pnts = list(map(list, zip(*pnts))) # transpose

ax.scatter(*pnts, s=100, marker='.', color='g', depthshade=False)

# plot fluxes and throats (red)

for throat in flux:

p1 = throat.pore1.pos; p2 = throat.pore2.pos

ax.plot([p1[0], p2[0]], [p1[1], p2[1]], [p1[2], p2[2]],

linewidth=max(0.5,abs(flux[throat]*2e6)), color='r', solid_capstyle='round')

Out:

**Figure:**
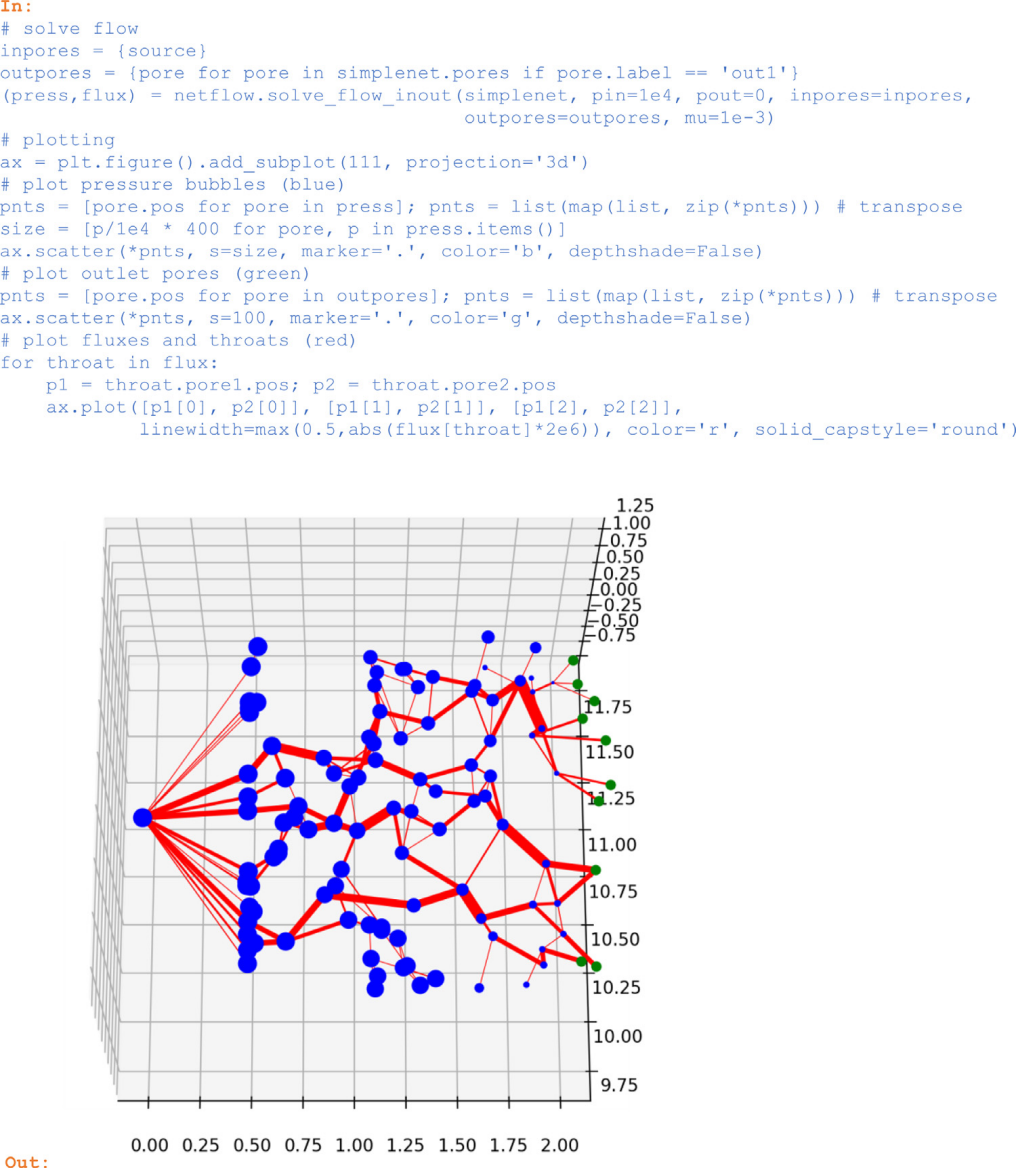

With *flux_balance*, for each pore or network node the balance of incoming and outgoing fluxes can be verified:

In:

Qb = netflow.flux_balance(press, flux)

print('the total inflow into the network is %e' % Qb[source][2])

# exclude pores in in-/outflow pore sets

Qb = {pore:Qb[pore] for pore in Qb if (pore not in inpores) and (pore not in outpores)}

x = range(len(Qb))

Qp = [Qb[k][0] for k in Qb]; Qm = [Qb[k][1] for k in Qb]; Qs = [Qb[k][2] for k in Qb]

plt.plot(x,Qp,x,Qm,x,Qs)

plt.gca().set_xlabel('pore index (without in-/outpores)')

plt.legend(['outgoing','incoming','sum'])

Out:

the total inflow into the network is 9.454177e-06

**Figure:**
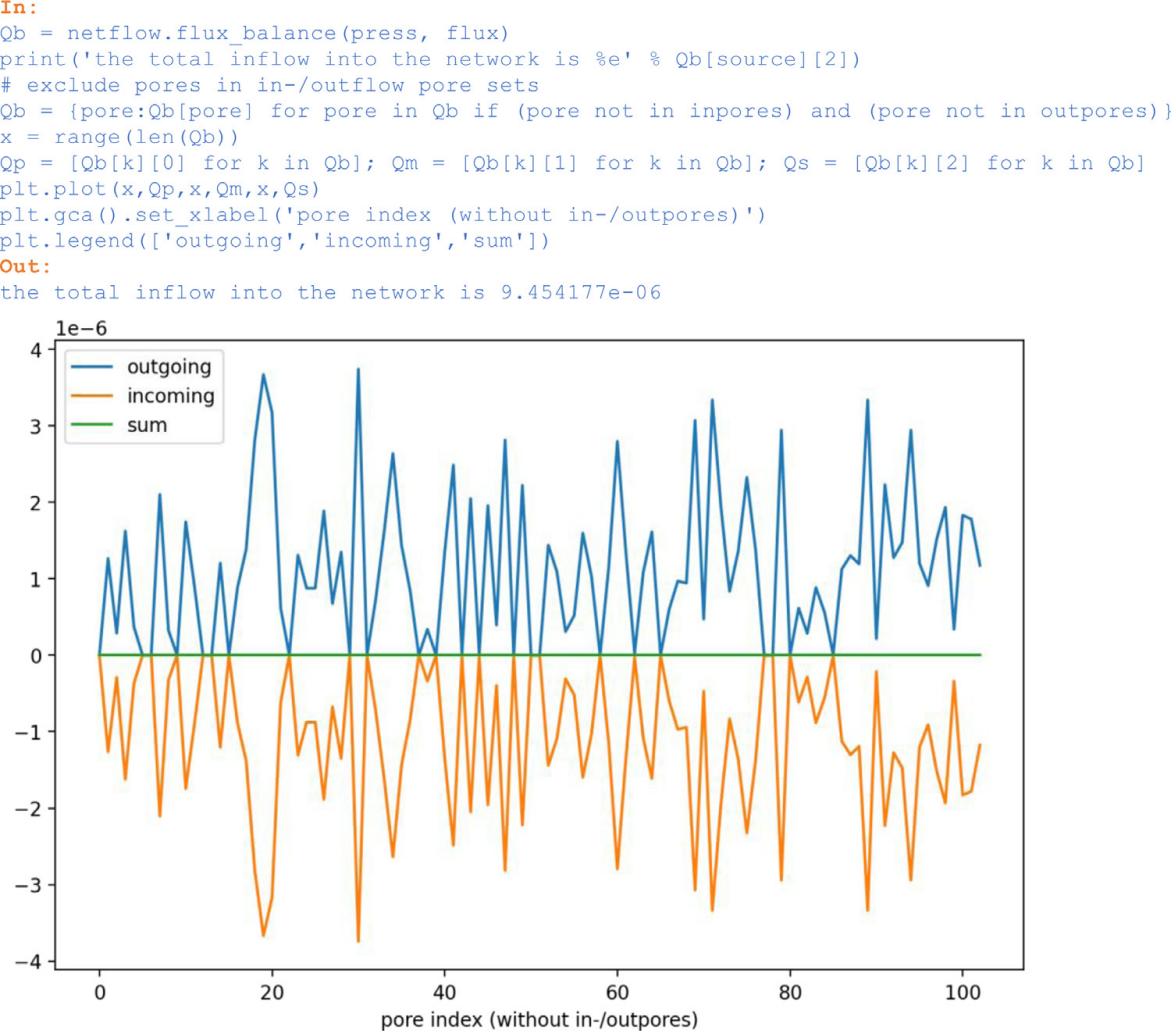

For periodic networks, the flow problem is solved with *solve_flow_periodic* by postulating periodicity in a fluctuating pressure part and by prescribing a driving mean pressure drop or gradient *P2L* in a prescribed *c*-direction. The total pore pressure results then from adding the mean and fluctuating pressure parts. More details about the flow problem in periodic networks are provided in section 2.2 of [2]. For the random network derived above from the demo network, the pressure solution looks as follows:

In:

mu = 1e-3 # dynamic viscosity [kg/(m*s)]

P2L = 1e2 # pressure drop [Pa/m = kg/(m*s^2)/m]

c = 3 # spatial direction of pressure drop

(press,flux) = netflow.solve_flow_periodic(dendronet, mu=mu, c=c, P2L=P2L)

# total pressure = fluctuating + mean pressure

for pore in press:

press[pore] = press[pore] - P2L*(pore.pos[c-1] - dendronet.lb[c-1])

# plot pore pressure bubbles

ax = plt.figure().add_subplot(111, projection='3d')

pnts = [pore.pos for pore in press]; pnts = list(map(list, zip(*pnts))) # transpose

col = [press[pore] for pore in press]; col = (max(col) - col) / (max(col) - min(col))

cmap = plt.cm.get_cmap('plasma')

col = [cmap(col) for col in col]

ax.scatter(*pnts, s=50, marker='.', color=col, depthshade=False)

# plot throats

for throat in flux:

if (throat.label == ''): # don't plot periodic throats

p1 = throat.pore1.pos; p2 = throat.pore2.pos

ax.plot([p1[0], p2[0]], [p1[1], p2[1]], [p1[2], p2[2]], linewidth=0.5, color='k')

**Figure:**
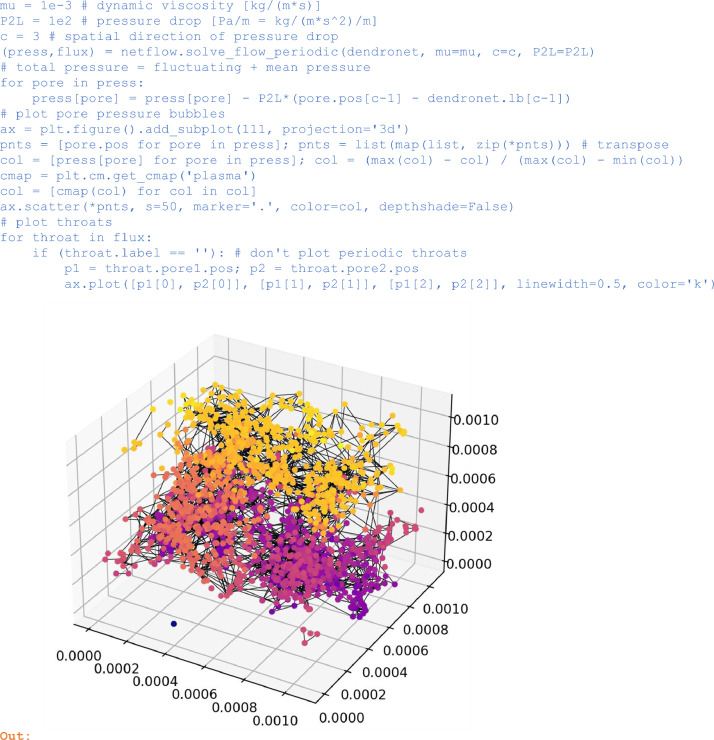

Out:

To save and restore flow solutions to and from hdf5 files, *save_flow_to* resp. *load_flow_from* can be used.

## Declaration of Competing Interest

None.

## Appendix Supplementary materials

Image, application 1
